# Current clinical evidence on pioglitazone pharmacogenomics

**DOI:** 10.3389/fphar.2013.00147

**Published:** 2013-11-26

**Authors:** Marina Kawaguchi-Suzuki, Reginald F. Frye

**Affiliations:** Department of Pharmacotherapy and Translational Research, Center for Pharmacogenomics, College of Pharmacy, University of FloridaGainesville, FL, USA

**Keywords:** pioglitazone, thiazolidinedione, CYP2C8, cytochrome P450, PPAR, pharmacokinetics, pharmacodynamics

## Abstract

Pioglitazone is the most widely used thiazolidinedione and acts as an insulin-sensitizer through activation of the Peroxisome Proliferator-Activated Receptor-γ (PPARγ). Pioglitazone is approved for use in the management of type 2 diabetes mellitus (T2DM), but its use in other therapeutic areas is increasing due to pleiotropic effects. In this hypothesis article, the current clinical evidence on pioglitazone pharmacogenomics is summarized and related to variability in pioglitazone response. How genetic variation in the human genome affects the pharmacokinetics and pharmacodynamics of pioglitazone was examined. For pharmacodynamic effects, hypoglycemic and anti-atherosclerotic effects, risks of fracture or edema, and the increase in body mass index in response to pioglitazone based on genotype were examined. The genes *CYP2C8* and *PPARG* are the most extensively studied to date and selected polymorphisms contribute to respective variability in pioglitazone pharmacokinetics and pharmacodynamics. We hypothesized that genetic variation in pioglitazone pathway genes contributes meaningfully to the clinically observed variability in drug response. To test the hypothesis that genetic variation in *PPARG* associates with variability in pioglitazone response, we conducted a meta-analysis to synthesize the currently available data on the *PPARG* p.Pro12Ala polymorphism. The results showed that *PPARG* 12Ala carriers had a more favorable change in fasting blood glucose from baseline as compared to patients with the wild-type Pro12Pro genotype (*p* = 0.018). Unfortunately, findings for many other genes lack replication in independent cohorts to confirm association; further studies are needed. Also, the biological functionality of these polymorphisms is unknown. Based on current evidence, we propose that pharmacogenomics may provide an important tool to individualize pioglitazone therapy and better optimize therapy in patients with T2DM or other conditions for which pioglitazone is being used.

## Introduction

Pioglitazone (PIO) is the most widely used thiazolidinedione (TZD) anti-diabetic drug. The first TZD, troglitazone, was approved for clinical use by the United States Food and Drug Administration (FDA) in 1997 (Kung and Henry, 2012). However, troglitazone was withdrawn from the market due to hepatotoxicity and is not currently available (Kung and Henry, 2012). PIO and rosiglitazone (ROSI) were approved by the FDA in 1999 (Kung and Henry, 2012). Unfortunately, due to an apparent increase in risk of myocardial infarction, ROSI was withdrawn from the European market in 2010; the FDA restricted the use of ROSI in the United States in 2011 (Kung and Henry, 2012). Therefore, PIO is currently the only TZD available without regulatory restrictions.

The exact mechanism of action of TZDs remains unclear, but the current consensus is that TZDs target the transcription factor peroxisome proliferator-activated receptor-γ (PPARγ) to improve insulin sensitivity (Cariou et al., 2012; Yau et al., 2013). Ligand-dependent transactivation of PPARγ causes heterodimerization with the retinoid-X receptor followed by recognition of peroxisome proliferator response elements (PPREs) that stimulate transcription of genes involved in metabolic homeostasis (Cariou et al., 2012). Additionally, PPARγ represses transcription of genes related to inflammation via transrepression, which does not require binding to PPREs but antagonizes other transcription factors that activate inflammatory pathways, such as NF-κ B and AP-1 (Cariou et al., 2012).

PIO was approved for the management of type 2 diabetes mellitus (T2DM) (Cariou et al., 2012; Yau et al., 2013). The efficacy of PIO as an anti-hyperglycemic agent was demonstrated in randomized controlled trials (Cariou et al., 2012). PIO monotherapy was shown to lower A1C by ~1% in T2DM patients (Yau et al., 2013). An advantage of PIO over sulfonylureas and insulin is that comparable A1C reduction is achieved without significant hypoglycemia (Yau et al., 2013). TZDs also improve insulin sensitivity, which may reduce insulin dose or eliminate the need for insulin in some patients (Yau et al., 2013). In addition to T2DM, PIO use has increased in other therapeutic areas such as non-alcoholic fatty liver disease, atherosclerosis, inflammation, infertility, and cancer due to the wide spectrum of effects secondary to PPARγ activation (Cariou et al., 2012; Yau et al., 2013). At the same time, more undesirable off-target effects have been identified. The use of PIO is typically limited by adverse drug reactions (ADRs) including bone fractures, peripheral edema, congestive heart failure (CHF), weight gain, and possible risk of bladder cancer (Cariou et al., 2012; Yau et al., 2013).

As more benefits and risks of PIO have been reported, clinically relevant variability in response has also been noted (Umpierrez and Dagogo-Jack, 2006; Yau et al., 2013). However, the sources of variability in response to PIO are not fully understood. In this article, we will address the question whether pharmacogenomics contributes to the observed variability in pioglitazone response. First, the current evidence on genetic variation in pioglitazone pathway genes will be summarized. Then, current evidence on genetic variation in the gene encoding the PPARγ receptor (*PPARG*) will be evaluated; because the data with PPARG are inconsistent, we conducted a meta-analysis to estimate the association between the best studied *PPARG* polymorphism and pioglitazone response. Finally, future research directions and methods to incorporate current pharmacogenomic findings in clinical practice are proposed.

## Methods

### Literature search

Two databases were used to identify pharmacogenomics evidence: PubMed and Web of Science. The searches were conducted through April 30, 2013. Two searches were performed with PubMed by using the following terms: (1) (“thiazolidinediones”[MeSH Terms] OR “thiazolidinediones”[All Fields]) AND (“pharmacogenetics”[MeSH Terms] OR “pharmacogenetics”[All Fields]) and (2) (“thiazolidinediones”[MeSH Terms] OR “thiazolidinediones”[All Fields]) AND (“polymorphism, genetic”[MeSH Terms] OR (“polymorphism”[All Fields] AND “genetic”[All Fields]) OR “genetic polymorphism”[All Fields] OR “polymorphism”[All Fields]). The numbers of articles found from the two searches were 33 and 91, respectively without any restrictions. The following searches were conducted with Web of Science: Topic = (pioglitazone) AND [Topic = (pharmacogenomics) OR Topic = (pharmacogenetics) OR Topic = (polymorphism)]. The original hit number was 84. The titles and abstracts were screened to include only clinical studies that investigated differences in PK or PD of PIO based on genetic polymorphisms in human participants. Articles only written in English were examined and meeting abstracts were not included in the summary. Sixteen full-text original research articles meeting our criteria and published in peer-reviewed journals were identified by our literature search.

### Meta-analysis

A meta-analysis was conducted with five identified studies examining the *PPARG* p.Pro12Ala polymorphism. The change in fasting plasma glucose (FG) from baseline was used as the primary outcome of interest because the definition of the responder phenotype was not consistent between studies. The data presented in the original articles were used. For the study by Bluher et al. (2003), the values were not available in the published article, so they were obtained directly from the authors [p.Pro12Pro −3.01 ± 3.53 mmol/L vs. p.Pro12Ala −2.98 ± 3.16 mmol/L (mean ± standard deviation)]. Units were converted from mmol/L to mg/dL to analyze changes in FG consistently among studies. Standard differences in means were used to compare the outcome of interest between the *PPARG* p.Pro12Pro genotype and 12Ala carriers by using the Comprehensive Meta-Analysis V2 program (Biostat, Englewood, NJ, USA). A test for heterogeneity (*I*^2^ = 48.7; *p* = 0.10) was conducted, and a fixed-effects model was used. The publication bias was checked (Supplemental figure 1).

## Results/discussions

### Effects of genetic variation on PIO pharmacokinetics

**Figure 1:**
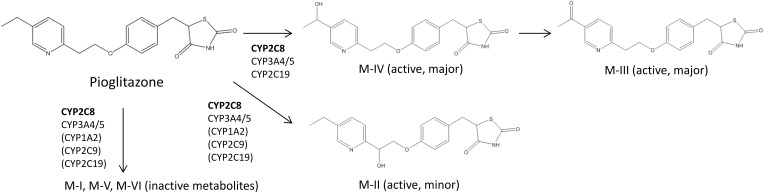
**Abbreviated Pioglitazone Metabolism Pathway.** CYP2C8 is the major enzyme metabolizing pioglitazone and is shown in bold. Enzymes in the parenthesis are suggested to be involved in pioglitazone metabolism, but their roles in the formation of particular metabolites are not clear according to currently available data (Eckland and Danhof, 2000; Jaakkola et al., 2006; Lai et al., 2009).

Table 1 summarizes clinical studies that investigated the effect of genetic polymorphisms on PIO pharmacokinetics. The primary metabolites of PIO are designated as M-I, M-II, M-IV, M-V, and M-VI (Eckland and Danhof, 2000) (Figure 1). M-IV is further metabolized to M-III and M-VI may also be formed from M-V (Eckland and Danhof, 2000). M-IV and M-III are known to be the major active metabolites and are responsible for the extended hypoglycemic effect (Eckland and Danhof, 2000). Multiple cytochrome P450 (CYP) enzymes are involved in the metabolism of PIO. However, CYP2C8 and CYP3A4 are the most important enzymes and contribute to ~60% and less than 20% of total PIO metabolism, respectively (Eckland and Danhof, 2000; Scheen, 2007; Vandenbrink et al., 2011).

**Table 1:** **Effect of genetic polymorphisms on pioglitazone pharmacokinetics**.

**Study**	**PIO (single dose)**	**Study population**	**Gene/polymorphism**	**Outcome**	**Results**
Aquilante et al., 2013	PIO 15 mg	•30 healthy Caucasians	*CYP2C8* *1/*1: *n* = 15 *1/*3: *n* = 14 ^*^3/^*^3: *n* = 1	AUC_0−∞_	*1/^*^1: 6770 ± 2480 ng^*^h/mL ^*^3 carriers: 4760 ± 2900 ng^*^h/mL ^*^3 carriers had 29.7% lower AUC_0−∞_ (*p* = 0.01) than *1 homozygotes
				AUC_0−∞_ with gemfibrozil (CYP2C8 inhibitor) 600 mg BID × 4 days	*1/^*^1: 21.100 ± 78.0 ng^*^h/mL ^*^3 carriers: 222.0 ± 99.0 ng^*^h/mL AUC_0−∞_ increased with gemfibrozil by 5.2-fold in ^*^3 carriers vs. 3.3-fold increase in ^*^1 homozygotes (*p* = 0.02)
Tornio et al., 2008	PIO 15 mg	•16 healthy volunteers	*CYP2C8* *1/^*^1: *n* = 8 *1/^*^3: *n* = 5 ^*^3/^*^3: *n* = 3	AUC_0−∞_ (weight-adjusted to 70 kg)	*1/^*^1: 4.95 ± 0.96 mg^*^h/L *1/^*^3: 3.67 ± 0.92 mg^*^h/L; 26% smaller than *1/^*^1 (*p* < 0.05) ^*^3/^*^3: 3.25 ± 0.87 mg^*^h/L; 34% smaller than *1/^*^1 (*p* < 0.05)
				AUC_0−∞_ (weight-adjusted to 70 kg) with trimethoprim (CYP2C8 inhibitor) 160 mg BID × 6 days	*1/^*^1: 6.60 ± 1.47 mg^*^h/L *1/^*^3: 5.70 ± 1.62 mg^*^h/L ^*^3/^*^3: 4.45 ± 0.46 mg^*^h/L (*p* < 0.05 vs. *1/^*^1) % change from PIO alone was *1/^*^1 133%, *1/^*^3 155%, and ^*^3/^*^3 137% (*p* = 0.017 between genotypes)
Kalliokoski et al., 2008	PIO 15 mg	•32 healthy Caucasians	*SLCO1B1* c.521T > C TT: *n* = 16 TC: *n* = 12 CC: *n* = 4	AUC_0−∞_ (weight-adjusted to 70 kg)	TT: 6422 ± 2050 ng^*^h/mL TC: 4922 ± 1062 ng^*^h/mL CC: 5384 ± 1469 ng^*^h/mL No significant effect on PK of PIO or the metabolites (M-III, M-IV, and M-V)

*TZD, thiazolidinedione; PIO, pioglitazone; AUC, area under the concentration-time curve; BID, twice daily; PK, pharmacokinetics; kg, kilograms*.

Two clinical studies examined genotypes of *CYP2C8*, the gene coding for the CYP2C8 enzyme. Both studies showed that compared to ^*^1 homozygotes, the ^*^3 variant allele carriers had ~30% lower total systemic exposure to PIO measured as the area under the plasma concentration-time curve (AUC) (Tornio et al., 2008; Aquilante et al., 2013). CYP2C8^*^3 is the best studied functional polymorphism in this gene. The ^*^3 allele is designated by the presence of two non-synonymous polymorphisms, rs11572080:G>A in exon 3 (p.Arg139Lys) and rs10509681:A>G in exon 8 (p.Lys399Arg) (Aquilante et al., 2013). The functional relevance of these polymorphisms appears to be substrate-dependent. For example, metabolism of PIO, ROSI, and repaglinide was increased, but metabolism of R-ibuprofen was decreased in carriers of the CYP2C8^*^3 allele (Martinez et al., 2005; Daily and Aquilante, 2009; Aquilante et al., 2013). The substrate selectivity of the enzyme is likely due to the amino acid changes in the protein structure caused by the polymorphisms. According to a recent population pharmacokinetic model, the oral clearance of PIO was ~52% higher in CYP2C8^*^3 carriers (Kadam et al., 2013). Considering that 10–23% of Caucasians carry the ^*^3 allele and that PIO exposure is decreased in carriers (Aquilante et al., 2013), this genotype is expected to explain some of the variability in response to PIO therapy. CYP2C8^*^3 is most frequently observed among Caucasians, followed by Hispanics, and the ^*^3 variant is rare among people with African and Asian ancestry (Aquilante et al., 2013; Martis et al., 2013).

The clinical studies also examined the influence of CYP2C8^*^3 on the magnitude of drug-drug interactions. Gemfibrozil was administered as a CYP2C8 inhibitor and demonstrated that the resulting increase in AUC was significantly greater in ^*^3 carriers (5.2-fold) compared with ^*^1 homozygotes (3.3-fold) (Aquilante et al., 2013). This resulted in PIO exposure after inhibition being similar in the two genotype groups (Aquilante et al., 2013). When trimethoprim was administered as a CYP2C8 inhibitor, the increase in AUC was larger in ^*^3 carriers, leading to a non-significant difference in the PIO exposure between ^*^1 homozygotes and ^*^1/^*^3 heterozygotes after the inhibition (Tornio et al., 2008). A similar genotype-dependent effect on the magnitude of drug-drug interaction has been reported with other CYP enzymes, such as CYP2C9, whereby the magnitude of interaction is greater in the individuals having the genotype associated with higher activity or clearance (Castellan et al., 2013). If a therapy known to inhibit CYP2C8 is initiated in patients with the ^*^3 allele taking PIO, close monitoring of PIO therapy is warranted because the inhibition may be more pronounced in this patient population.

The effect of genetic variation in the gene that encodes the organic anion-transporting poly-peptide OATP1B1 (*SLCO1B1*, Solute carrier organic anion transporter family member 1B1) has also been studied. OATP1B1 is an uptake transporter located at the basolateral membrane of hepatocytes that facilitates drug entry into the hepatocyte (Zamek-Gliszczynski et al., 2012). PIO is a substrate and potent competitive inhibitor of OATP1B1 (Kalliokoski et al., 2008). However, no significant effect of *SLCO1B1* polymorphisms on the PK of PIO and its metabolites (M-III, M-IV, and M-V) was observed (Kalliokoski et al., 2008). Because the results have not yet been replicated, transporter polymorphisms should be further studied with PIO.

### Effects of genetic variation on PIO pharmacodynamics

Table 2 summarizes studies that examined the extent to which genetic polymorphisms affect PIO pharmacodynamics.

**Table 2:** **Effect of genetic polymorphisms on pioglitazone pharmacodynamics**.

**Study**	**TZD regimen**	**Study population (inclusion criteria)**	**Gene/polymorphism**	**Outcome**	**Results**
Bluher et al., 2003	PIO 45 mg QD for >26 weeks	131 patients with T2DM A1C = 7.5–11.5% FG = 7.8–14.0 mmol/L BMI = 25–35 kg/m^2^ No other antidiabetic medications	PPARG Pro12Ala p.Pro12Pro = 110 p.Pro12Ala = 16 p.Ala12Ala = 5	>20% decrease in FG	p.Pro12Pro: OR 0.45; *p* = 0.67 p.Pro12Ala: OR 0.78; *p* = 0.31 p.Ala12Ala: OR 0.61; *p* = 0.76
				15% decrease in A1C	p.Pro12Pro: OR 0.66; *p* = 0.23 p.Pro12Ala: OR 0.42; *p* = 0.2 p.Ala12Ala: OR 0.48; *p* = 0.61
Ramirez-Salazar et al., 2008	PIO 45 mg QD for 15 days	77 obese menopausal women in Mexico BMI >30 kg/m^2^	PPARG Pro12Ala p.Pro12Pro = 59 p.Pro12Ala = 18	Change in FG	p.Pro12Pro: −7 ± 8 mg/dL p.Pro12Ala: −15 ± 15 mg/dL *p* < 0.003
				HOMA-IR	p.Pro12Pro: −1 (−2.5 to 0.07) p.Pro12Ala: −0.08 (−0.68 to 1.05) *p* < 0.03, mean (25th–75th quartiles)
Hsieh et al., 2010	PIO 30 mg QD for 24 weeks	250 Chinese patients with T2DM A1C = 7–11% FG = 130–250 mg/dL BMI = 25–35 kg/m^2^ No change in medications in the previous 3 months	PPARG Pro12Ala p.Pro12Pro = 197 p.Pro12Ala = 53	15% decrease in A1C or 20% decrease in FG	p.Pro12Pro: 57.9% p.Pro12Ala: 75.5% OR 2.39; 95% CI 1.13–5.03; *p* = 0.022
			PPARGC1A p.Gly482Ser p.Gly482Gly = 51 p.Gly482Ser = 199	15% decrease in A1C or 20% decrease in FG	p.Gly482Gly: 58.8% p.Gly482Ser: 62.3% OR 1.17; 95% CI 0.58–2.36; *p* = 0.66
Namvaran et al., 2011	PIO 15 mg QD for 12 weeks	101 Iranian patients with T2DM No change in previous medications	PPARG p.Pro12Ala p.Pro12Pro = 95 p.Pro12Ala = 6	15% decrease in A1C	p.Pro12Pro: 31.6% p.Pro12Ala: 33.3% NS
Pei et al., 2013	PIO 30 mg QD for 3 months	67 Chinese patients with T2DM BMI = 19–30 kg/m^2^ No other insulin secretagogue No change in medications in the previous 3 months *CYP2C8* ^*^1/^*^1 genotype	PPARG rs1801282 (p.Pro12Ala) CC = 60 CG = 7	Change in FG	CC: −1.23 ± 0.48 mmol/L CG: −2.24 ± 8.2 mmol/L *P* < 0.05
			PTPRD rs17584499 CC = 45 CT + TT = 22	Change in PPG	CC: −3.18 ± 3.37 mmol/L CT + TT: −0.63 ± 3.26 mmol/L *P* < 0.01
Li et al., 2008	PIO 30 mg QD × 10 weeks	113 Chinese patients with T2DM A1C >7% FG >7 mmol/L No change in previous medications	ADIPOQ C-11377G CC = 58 CG + GG = 55	Change in FG	CC: −0.22 ± 0.16 mmol/L CG + GG: −0.26 ± 0.19 mmol/L *p* = 0.201
				Change in A1C	CC: −0.08 ± 0.11% CG + GG: −0.13 ± 0.13% *P* = 0.028
			ADIPOQ G-10068A GG = 65 GA + AA = 48	Change in FG	GG: −0.25 ± 0.15 mmol/L GA + AA: −0.23 ± 0.21 mmol/L *p* = 0.593
				Change in A1C	GG: −0.11 ± 0.12% GA + AA: −0.10 ± 0.12% *p* = 0.811
			ADIPOQ A-4041C AA = 48 AC + CC = 25	Change in FG	AA: −0.25 ± 0.17 mmol/L AC + CC: −0.25 ± 0.19 mmol/L *p* = 0.792
				Change in A1C	AA: −0.12 ± 0.11% AC + CC: −0.11 ± 0.10% *p* = 0.398
			ADIPOQ T45G TT = 65 TG + GG = 48	Change in FG	TT: −0.23 ± 0.20 mmol/L TG + GG: −0.25 ± 0.16 mmol/L *p* = 0.585
				Change in A1C	TT: −0.11 ± 0.13% TG + GG: −0.10 ± 0.10% *p* = 0.925
Namvaran et al., 2012	PIO 15 mg QD × 12 weeks	101 Iranian patients with T2DM A1C >7% FG >7 mmol/L No change in medications in the previous 3 months	ADIPOQ T45G (rs2241766) TT = 66% TG = 31% GG = 3%	15% decrease in A1C	TT: 34.3% TG + GG: 26.5% OR 1.85; 95% CI 0.72–4.76; *p* = 0.20
			ADIPOR2 G795A (rs16928751) GG = 70% GA = 20% AA = 10%	15% decrease in A1C	GG: 29.6% AG: 45% AA: 20% OR 0.97; 95% CI 0.38-2.50; *p* = 0.97
Makino et al., 2009	PIO 15 mg QD × 4 weeks followed by 30 mg × 8 weeks (or PIO 30 mg × 12 weeks)	121 Japanese patients with T2DM and 63 patients in the replication cohort A1C 6.5–12% BMI = 16–35 kg/m^2^ No change in medications in the previous 3 months	RETN C-420G (rs1862513) CC = 55 CG = 54 GG = 12	Change in FG	CC: −31.1 ± 33.2 mg/dL CG: −37.3 ± 32.8 mg/dL GG: −54.1 ± 34.6 mg/dL *p* = 0.116
			Replication cohort: CC = 30 CG = 27 GG = 6		Regression coefficient CG: −4.80 ± 6.38 mg/dL(*p* = 0.453, vs. CC) GG: −24.7 ± 10.6 mg/dL (*p* = 0.021, vs. CC)
				Change in A1C	CC: −0.9 ± 0.8% CG: −0.8 ± 0.7% GG: −0.9 ± 0.7% *p* = 0.832
					Replication cohort: CC: −1.3 ± 1.0% CG: −1.2 ± 1.3% GG: −2.7 ± 2.3% *p* = 0.033
Wang et al., 2007	PIO 30 mg × 10 weeks	113 Chinese patients with T2DM A1C =7–12% FG ≤16.9 mmol/L Other anti-hyperglycemic agents were allowed (no change in the previous 3 months)	LPL S447X SS 86.73% SX 12.39% XX 0.88% MAF = 7.08%	>10% decrease in FG	SS 84%, non-SS 60% OR 0.54; 95% CI 0.30–0.97; *p* = 0.04
				>1% decrease in A1C	SS 57%, non-SS 27% OR 0.74; 95% CI 0.42–1.30; *p* = 0.30
Saitou et al., 2010	PIO	62 Japanese patients with T2DM	ACE I/D in intron 16 MAF = 16.4%	IMT	NS
			MTHFR C677T MAF = 16.4%	IMT	NS
Himelfarb et al., 2011	PIO 15, 30, 45, and 45 mg QD each 4 weeks (total 16 weeks)	53 Brazilian patients with T2DM No other hypoglycemic agents or insulin	TNFA −308G>A (rs11800629) MAF = 16.4%	OGTT-2 h glucose	NS
				Bone biomarkers	The A variant allele was associated with lower tAPL levels, suggesting reduced osteoblastic activity after PIO therapy (*p* = 0.017)
			IL6 −174G>C (rs1800795) MAF = 13.9%	OGTT-2 h glucose	The minor allele was associated with decreased OGTT-2 h glucose levels (*p* < 0.05)
				Bone biomarkers	NS
Chang et al., 2011	PIO (*n* = 21) or ROSI (*n* = 247)	268 Taiwanese patients with T2DM	AQP2 rs296766 CC = 203 CT = 63 TT = 2	Edema	The T variant allele was associated with TZD-related peripheral edema. OR 2.89; 95% CI 1.61–5.17; *p* = 0.0059
			SLC12A1 rs12904216 AA = 122 AG = 106 GG = 40	Edema	GG genotype was associated with TZD-related peripheral edema OR 2.66; 95%CI 1.26–5.63; *p* = 0.011
Ruaño et al., 2009	PIO (*n* = 33) or ROSI (*n* = 54) for ≥4 months	87 patients with T2DM The use of other anti-diabetics was allowed	ADORA1 rs903361 MAF = 33.0%	BMI	Presence of the variant allele was associated with greater increase in BMI Regression coefficient 3.4; *p* = 0.0003; false discovery rate = 0.10

*TZD, thiazolidinedione; PIO, pioglitazone; T2DM, type 2 diabetes mellitus; QD, once daily; MAF, minor allele frequency; HOMA-IR, Homeostasis Model of Assessment-Insulin Resistance index; OGTT, oral glucose tolerance test; NS, not significant; IMT, carotid intima-media thickness; FG, fasting plasma glucose; PPG, postprandial plasma glucose; OR, odds ratio; 95% CI, 95% confidence interval; ROSI, rosiglitazone*.

#### Hypoglycemic effect

The most well-studied polymorphism with respect to PIO response is *PPARG* p.Pro12Ala (rs1801282). *PPARG* is the gene encoding PPARγ, a ligand-activated transcription factor that regulates glucose homeostasis (Auwerx, 1999). The minor allele frequency for this polymorphism has been reported as 2–18% in healthy humans (Saraf et al., 2012). The 12Ala allele appears to improve insulin sensitivity due to an alteration in the transcriptional activity of PPARγ (Deeb et al., 1998; Saraf et al., 2012). This variant has been consistently associated with decreased risk of T2DM in previous studies (Altshuler et al., 2000). According to a meta-analysis, the odds ratio of T2DM risk was 0.86 (95% confidence interval 0.81–0.90) with the presence of the 12Ala allele (Gouda et al., 2010).

PPARγ is the drug target of TZDs, thus it would be biologically plausible for the functional p.Pro12Ala polymorphism in the *PPARG* gene to be associated with response to TZDs. Previously, in response to ROSI, *PPARG* p.Pro12Ala allele carriers achieved greater reduction in FG (50.6 ± 27.8 vs. 24.3 ± 41.9 mg/dL, *p* = 0.026) and in A1C (1.41 ± 1.47% vs. 0.57 ± 1.16%, *p* = 0.015) than patients without the variant allele (Kang et al., 2005).

**Figure 2:**
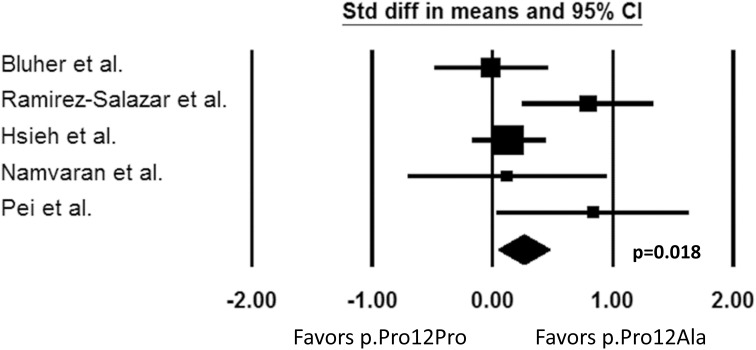
**Change in fasting plasma glucose from baseline.** Comparison of *PPARG* p.Pro12Pro genotype vs. p.Pro12Ala carriers (Std diff in means, standard difference in means; 95% CI, 95% confidence interval).

Our search identified five pharmacogenetic studies in which the effect of *PPARG* p.Pro12Ala polymorphism on PIO pharmacodynamic response was examined. The results were not entirely consistent across studies. Three out of five studies demonstrated a better response in terms of improvements in FG and A1C in carriers of the 12Ala allele (Ramirez-Salazar et al., 2008; Hsieh et al., 2010; Pei et al., 2013). However, two studies reported a non-significant response to PIO (Bluher et al., 2003; Namvaran et al., 2011). It is difficult to explain exactly why the results were disparate, but possible reasons include the heterogeneity of the studies and small sample sizes of patients with the minor allele. The studies were quite heterogeneous with respect to the patient population, the inclusion/exclusion criteria, PIO treatment regimen, and the presence of other anti-diabetic treatments. In addition, the two negative studies had a relatively small number of patients with the 12Ala minor allele, suggesting power may not have been adequate to detect a difference. Although the results are conflicting, the *PPARG* p.Pro12Ala polymorphism may contribute to the variability observed in response to PIO as shown in the three favorable studies. Indeed, according to our meta-analysis of the five studies identified, *PPARG* 12Ala allele carriers had a more favorable change in FG compared to people with the p.Pro12Pro genotype (*p* = 0.018, Figure 2). However, this polymorphism alone does not fully explain why some patients failed to respond to PIO. Other genetic and environmental factors are likely to be involved and may have contributed to the negative results in the two studies. Thus, consideration of other genes appears important to better explain the variability in PIO treatment response.

In addition to the *PPARG* gene, protein tyrosine phosphatase receptor type D (*PTPRD*) gene was investigated. *PTPRD* polymorphisms associated with T2DM in a genome wide association study in a Chinese Han population (Tsai et al., 2010). Rs17584499, located in intron 10, was the single nucleotide polymorphism (SNP) with the strongest association (Tsai et al., 2010). The functionality of this polymorphism has not yet been identified. Considering that it is located in an intron, the polymorphism may affect mRNA splicing or may be in linkage disequilibrium with other functional polymorphisms. One study found that patients with rs17584499 wild-type CC genotype had better postprandial plasma glucose concentrations after being treated with PIO for 3 months compared with T minor allele carriers (Pei et al., 2013). Confirmation of these results is needed in an independent cohort. However, it is clear that more polymorphisms remain to be uncovered to better explain the variability in PIO response. Such polymorphisms may be identified through populations that have been previously under-represented.

The adiponectin (*ADIPOQ*) gene has been studied in patients receiving PIO therapy. Adiponectin is an adipokine, a peptide hormone secreted from adipocytes, known to play an important role in insulin sensitization and fat β-oxidation (Li et al., 2008; Ruscica et al., 2012). T2DM and insulin resistance have been associated with low serum adiponectin levels (Li et al., 2008). Multiple polymorphisms in *ADIPOQ* associated with T2DM in various ethnic populations, including Asians and Caucasians with European ancestry (Hara et al., 2002; Menzaghi et al., 2002; Stumvoll et al., 2002; Vasseur et al., 2002, 2003; Vozarova De Courten et al., 2005; Woo et al., 2006; Li et al., 2008; Jing et al., 2012).

Two studies investigating response to PIO with respect to *ADIPOQ* genotypes were identified in the literature search. These studies were conducted in Chinese and Iranian populations (Li et al., 2008; Namvaran et al., 2012). However, in the two studies, the only significant change in FG or A1C in response to PIO was found with *ADIPOQ* C-11377G genotype in the study of the Chinese population (Li et al., 2008; Namvaran et al., 2012). The −11377 CC genotype patients had smaller reduction in A1C than the minor G allele carriers (Li et al., 2008). Response to ROSI was also previously studied with regard to the *ADIPOQ* polymorphism. In Chinese T2DM patients treated with ROSI, the wild-type −11377 CC genotype was associated with greater reduction in FG, compared to the CG and GG genotypes (−34.2 vs. −9.7 mmol/L, *p* = 0.001) (Sun et al., 2008). A definitive conclusion on this *ADIPOQ* polymorphism cannot be made due to the lack of replication and because the direction of the association was opposite with the *ADIPOQ* C-11377G genotype between the ROSI and PIO studies. Studies have shown that exercise affects adiponectin concentrations (Gueugnon et al., 2012; Lee et al., 2013). The Korean study reported that exercise increased adiponectin levels irrespective of *ADIPOQ* polymorphisms (Lee et al., 2013). Therefore, it may be possible that environmental factors, such as exercise level, have a large impact and make it difficult to differentiate the effect of *ADIPOQ* polymorphisms on response to PIO.

Adiponectin receptor 2 (*ADIPOR2*) is another gene studied along with *ADIPOQ* polymorphism to explain PIO response variability. The *ADIPOR2* gene is highly expressed at critical sites involved in glucose metabolism and activation of this receptor is known to increase fatty acid oxidation and adiponectin-mediated glucose utilization (Kadowaki et al., 2006; Namvaran et al., 2012). However, in an Iranian population, *ADIPOR2* G795A genotype did not have any significant effect on FG and A1C in response to PIO (Namvaran et al., 2012). No definitive conclusions can be made from one study and the functionality of *ADIPOR2* polymorphisms needs to be investigated further to understand the involvement in PIO response. However, if any polymorphism in *ADIPOR2* can affect the functionality of the receptor, it may still be possible that the polymorphism could have an influence on response to PIO therapy.

The involvement of the resistin (*RETN*) gene in response to PIO was studied in a Japanese population. Resistin is another adipokine known to antagonize insulin (Osawa et al., 2004). The role of *RETN* polymorphisms in T2DM or obese patients has not been fully elucidated, but the *RETN* C-420G (rs1862513) GG genotype was previously associated with T2DM susceptibility (Osawa et al., 2004; Ochi et al., 2007; Makino et al., 2009). Sp1 and Sp3 are transcription factors that bind to the DNA element, if the −420 G allele is present, to enhance serum resistin concentrations via increased *RETN* promoter activity (Osawa et al., 2004). The binding of Sp1/3 does not occur with the −420 C allele (Osawa et al., 2004). One report suggested that TZD-mediated PPARγ activation represses the expression of the resistin gene by modulating Sp1 activity (Chung et al., 2005; Makino et al., 2009). Makino et al. studied *RETN* C-420G polymorphism in Japanese T2DM patients and found that the GG genotype was associated with better FG and A1C reduction (Makino et al., 2009). However, the reduction in A1C was observed only in the replication cohort, and the significance with the FG reduction did not remain after adjustment for multiple testing. The authors commented on differences in the patient characteristics between the original and replication cohorts and that the sample size might not have been adequate. It is reasonable to hypothesize that patients with the GG genotype can recruit more Sp1 at baseline and therefore have more pronounced reduction in serum resistin levels with PIO treatment. However, the effect of this *RETN* C-420G SNP on PIO response needs to be further investigated with the involvement of Sp1/3 signaling pathway.

The lipoprotein lipase (*LPL*) S447X polymorphism has been studied with respect to PIO response. Lipoprotein lipase is the rate-limiting enzyme for the breakdown of lipoproteins rich in triglycerides (Groenemeijer et al., 1997). Lipoprotein lipase is synthesized by and secreted from various parenchyma cells including adipocytes, muscle cells, and macrophages and expression was increased by PIO in a previous *in vivo* study (Bogacka et al., 2004; Wang et al., 2007). Among more than 40 polymorphisms in *LPL* gene, S447X is the most extensively studied polymorphism; it causes a premature stop codon and the elimination of serine and glycine at the C-terminal (Wang et al., 2007). Previous independent studies reported an association of the S447X variant with dyslipoproteinemia and coronary artery disease (Kuivenhoven et al., 1997; Wang et al., 2007). This variant was investigated in T2DM patients who received PIO and found that the response rate (defined as >10% reduction in FG) to PIO was 0.54-fold smaller in patients with the *LPL* S447X genotype, compared to patients with the S447S genotype (Wang et al., 2007). Although independent confirmation of this result is needed, *LPL* is another candidate gene to be studied with response to PIO. In addition, more functional studies are necessary to understand how this polymorphism relates to glycemic control in humans.

#### Anti-atherosclerotic effect

The effect of genetic polymorphism on carotid atherosclerosis progression has been studied in patients who received PIO. TZDs were shown to improve lipid profiles, inflammation, coagulation, and endothelial cell function, and PIO was shown to significantly slow progression of carotid intima-media thickness (IMT) (Saitou et al., 2010). In contrast to ROSI, which was associated with an increased risk of MI, meta-analyses showed that PIO did not increase the risk of MI and might have a benefit of decreasing revascularization events (Nagajothi et al., 2008; Tannen et al., 2013). A study investigated 99 candidate gene polymorphisms, which were previously associated with atherosclerosis, diabetes mellitus, hypertension, or dyslipidemia, among T2DM patients treated with PIO (Saitou et al., 2010). Results for the angiotensin-converting enzyme (*ACE*) gene and methylene-tetrahydrofolate reductase (*MTHFR*) gene were reported, but the IMT was not significantly affected by polymorphisms in these two genes (Saitou et al., 2010). However, they compared the results with patients treated with diet alone. The IMT significantly increased in *ACE* D allele carriers (average IMT from 0.802 ± 0.017 to 0.831 ± 0.017 mm, *p* = 0.006; max IMT from 0.969 ± 0.023 to 0.998 ± 0.021 mm, *p* = 0.05) and the *MTHER* 677T allele carriers (average IMT from 0.804 ± 0.012 to 0.839 ± 0.013 mm, *p* = 0.001; max IMT from 0.969 ± 0.023 to 1.010 ± 0.018 mm, *p* = 0.007) (Saitou et al., 2010). Thus, the study demonstrated that PIO attenuated the increase in IMT irrespective of the genotypes.

#### Fracture risk

There is much concern with bone fractures as an ADR with TZDs. Both ROSI and PIO are known to provoke bone loss, and increased risks of fractures are reported, especially among women with T2DM taking a TZD (Betteridge, 2011). The most common sites of fractures are at distal limbs rather than at hips and spines even though the reason for this localization of fractures is still unclear (Betteridge, 2011; Cariou et al., 2012). PPARγ-mediated actions on bone homeostasis are believed to be responsible for these fractures. The activation of PPARγ was shown to promote osteoclastogenesis and suppress osteoblastogenesis, leading to bone loss as the net effect (Wan, 2010). Tumor necrosis factor α (TNFα) and interleukin-6 (IL-6) are inflammatory cytokines, and their release is suppressed by PPARγ activation (Cariou et al., 2012). TNFα differentiates human peripheral monocytes into activated osteoclasts, so the reduced TNFα level can result in inhibition of osteoclastogenesis (Hounoki et al., 2008).

TNFα and IL-6 are encoded by *TNFA* and *IL6* genes respectively, and the effect of *TNFA* and *IL6* genotypes was studied in T2DM patients who received PIO (Himelfarb et al., 2011). They showed that *TNFA* –308A allele carriers had decreased mRNA expression compared with those with the wild GG genotype (Himelfarb et al., 2011). They also found that alkaline phosphatase, a biomarker for osteoblastic activity, was reduced in response to PIO in *TNFA* −308A allele carriers with T2DM (Himelfarb et al., 2011). *IL6* −174C allele was associated with decreased OGTT-2h glucose but not with any bone metabolic markers (Himelfarb et al., 2011). *IL6* −174C allele was previously associated with higher plasma levels of IL-6, but in this study, mRNA expression did not differ by the *IL6* genotype (Himelfarb et al., 2011). Since replication is lacking and studies relating the genotypes directly to the occurrence of fractures are missing, no definitive conclusion can be made at this point.

#### Edema risk

Edema is a potential ADR from ROSI and PIO, and both TZDs carry a FDA black box warning for CHF (Kung and Henry, 2012). The safety of TZDs for patients with New York Heart Association class III or IV CHF is not established, and the use of TZDs is contraindicated in this patient population (Kung and Henry, 2012; Yau et al., 2013). The underlying mechanism of both edema and CHF appears to be fluid retention and expansion of plasma volume (Kung and Henry, 2012). Two studies that analyzed the TZD-related edema risk were identified in the literature search.

Twenty eight SNPs were genotyped from genes related to sodium and water reabsorption (Chang et al., 2011). They found that the *AQP2* rs296766 T allele and *SLC12A1* rs12904216 GG genotype were associated with edema in T2DM patients treated with a TZD (Chang et al., 2011). *AQP2* gene codes aquaporins, which function as a water channel on the apical membrane, predominantly in the collecting duct of the kidney (Knepper et al., 1996). The *AQP2* rs296766 SNP is located at the 3' untranslated region and may regulate mRNA stability (Chang et al., 2011). *SLC12A1* encodes apical Na-K-2CL cotransporter, NKCC2, which plays an essential role in concentrating urine with the uptake of CL^−^ and K^+^ driven by the Na^+^ influx (Ji et al., 2008; Chang et al., 2011). This transporter, coded by *SLC12A1*, is a target of thiazides and furosemide, and genetic polymorphisms in this gene were associated with blood pressure variation, Bartter syndrome, and response to loop diuretics (Ji et al., 2008; Chang et al., 2011). The *SLC12A1* rs12904216 SNP is located in intron 7, and the protein expression may be altered by this SNP (Chang et al., 2011). Further studies are necessary to replicate the association, to describe how *AQP2* rs296766 and *SLC12A1* rs12904216 SNPs affect the coded transporter function, and to clarify a possible existence of other functional polymorphisms in linkage disequilibrium.

The other study genotyped 384 SNPs in genes involved in cardiovascular and metabolic pathways (Ruaño et al., 2009). The top five polymorphisms possibly associated with edema were: neuropeptide Y (*NPY*) rs1468271 (*p* = 0.006), glycogen synthase 1 (*GYS1*) rs2287754 (*p* = 0.013), chemokine C-C motif ligand 2 (*CCL2*) rs3760396 (*p* = 0.015), oxidized low density lipoprotein receptor 1 (*OLR1*) rs2742115 (*p* = 0.015), and growth hormone releasing hormone (*GHRH*) rs6032470 (*p* = 0.023) (Ruaño et al., 2009). However, none of the polymorphisms associated with edema after accounting for multiple comparisons.

The other factors related to edema were women sex and older age (Chang et al., 2011). According to these findings and the pharmacogenetic data, the investigators developed a simple point system to predict the likelihood of TZD-related edema based on age, sex, and *AQP2* rs296766 and *SLC12A1* rs12904216 genotypes (Chang et al., 2011). Although this point system needs to be validated in an independent cohort, the development of a simple scoring system is a good example of making pharmacogenetic information more useful and meaningful in clinical practice.

#### Increase in body mass index (BMI)

Besides the occurrence of edema, Ruaño et al. (2009) investigated possible associations of the 384 SNPs from cardiovascular and metabolic pathways with change in BMI. Weight gain is one of the common ADRs of TZDs. In a PIO monotherapy trial, patients gained 2.82 kg after the treatment of 45 mg PIO for 26 weeks while weight loss was observed in a placebo group (Kung and Henry, 2012). The weight gain appears to be dose-dependent, and the mechanism is not only related to the fluid retention but also increase in subcutaneous fat depots (Kung and Henry, 2012). The top five polymorphisms possibly associated with change in BMI were: adenosine A1 receptor (*ADORA1*) rs903361 (0.0003), pyruvate kinase (*PKM2*) rs2856929 (*p* = 0.002), *ADIPOR2* rs7975375 (*p* = 0.007), uncoupling protein 2 (*UCP2*) rs660339 (*p* = 0.008), and apolipoprotein H (*APOH*) rs8178847 (*p* = 0.010) (Ruaño et al., 2009). After adjustment for multiple comparisons, rs903361, a SNP located in intron 2 of the *ADORA1* gene, was significantly associated with increase in BMI (Ruaño et al., 2009). The adenosine A1 receptor is a G-protein-coupled receptor highly expressed in adipose tissue and activation of this receptor leads to inhibition of lipolysis (Dhalla et al., 2009). How the rs903361 SNP affects the functionality of the receptor is unknown and the association of this SNP with BMI must still be replicated in an independent cohort.

## Conclusions

Clinically relevant variability in pioglitazone response has been demonstrated, but the underlying factors contributing to variability are not well-understood. This hypothesis article summarizes current evidence on pioglitazone pharmacogenomics; current data suggest that genetic variation is indeed an important factor contributing to pioglitazone response. Thus, we posit a strategy incorporating pharmacogenomic information may provide a more rational approach to achieve optimal outcomes. According to PharmGKB (https://www.pharmgkb.org), CYP2C8^*^3 is the only genetic variant annotated for PIO treatment as level 3 evidence, which was defined as “annotation for a variant-drug combination based on a single significant (not yet replicated) or annotation for a variant-drug combination evaluated in multiple studies but lacking clear evidence of an association”(Whirl-Carrillo et al., 2012). However, more studies investigating genetic effect on PIO therapy are now available. In this article, the PIO pharmacogenetic evidence to date is summarized. The genes investigated for sources of variability in PIO PK are *CYP2C8* and *SLCO1B1*. The variant allele CYP2C8^*^3 is associated with higher PIO clearance, which results in reduced exposure to PIO in carriers of this allele. The alteration in PIO PK has clinical implications, and the relevant consequences with drug interaction are a concern. Thus, if an inhibitor, inducer or substrate of CYP2C8 is to be administered with PIO in known CYP2C8^*^3 carriers, closer monitoring may be warranted. For future studies of PIO, *CYP2C8* genotyping is recommended to better understand the variability in response to PIO and to interpret data more clearly when CYP2C8^*^3 carriers are present in the study. This may be especially important because of racial differences in CYP2C8^*^3 allele frequency. The ^*^3 variant has been the focus of clinical PK studies with PIO, but the functionality of other *CYP2C8* variant alleles need to be further determined preferably *in vivo* because the discrepancy between *in vitro* and *in vivo* data have been shown with CYP2C8 activity. In contrast to *CYP2C8*, there is currently no evidence to suggest that genetic variation in drug transporters affects PIO disposition.

The genetic polymorphism most widely studied to better understand variability in PIO PD is *PPARG* p.Pro12Ala. T2DM patients with *PPARG* p.Pro12Ala variant are likely to have better glycemic response with PIO. Thus, the *PPARG* p.Pro12Ala is a promising polymorphism and one that should be included in clinical models developed to predict a patient's response based on genotype. However, this polymorphism alone is unlikely to explain all of the variability seen among patients. Consequently, other genetic variants as well as clinical factors must be considered. Indeed, several polymorphisms were identified in other genes related to PIO pathways. However, it is difficult to make any firm conclusions, based on single studies reporting a significant association, especially if it is not known how the polymorphism affects the functionality of the coded protein. Similarly, the identified polymorphism may merely be in linkage disequilibrium with the true functional variant. Therefore, the data must be interpreted with caution. Validation or replication of the finding is necessary before the pharmacogenetic knowledge can be applied to patient care. To better explain the variability in response, gene-gene and gene-environment interactions should also be considered.

Based on current evidence, we propose that pharmacogenomics may provide an important tool to individualize pioglitazone therapy. For example, developing a scoring system that incorporates both clinical and genetic factors may provide more clinical utility and make the pharmacogenetic evidence more accessible to clinicians who are unfamiliar with this field. This is important because the data from several genes suggest PIO pharmacogenomics has the potential to be an important means to better optimize therapy in patients with T2DM.

## Supplementary material

The Supplementary Material for this article can be found online at: http://www.frontiersin.org/journal/10.3389/fphar.2013.00147/abstract

### Conflict of interest statement

The authors declare that the research was conducted in the absence of any commercial or financial relationships that could be construed as a potential conflict of interest.

## Abbreviations

ACE: angiotensin-converting enzyme
ADIPOQ: adiponectin
ADIPOR: Adiponectin receptor
ADORA1: adenosine A1 receptor
ADR: adverse drug reaction
APOH: apolipoprotein H
AUC: area under the plasma concentration-time curve
BMI: body mass index
CCL2: chemokine C-C motif ligand 2
CHF: congestive heart failure
CYP: cytochrome P450
FDA: Food and Drug Administration
FG: fasting plasma glucose
GHRH: growth hormone releasing hormone
GYS1: glycogen synthase 1
IL-6: interleukin-6
IMT: intima-media thickness
LPL: lipoprotein lipase
MTHFR: methylene-tetrahydrofolate reductase
NPY: neuropeptide Y
OATP: organic anion transporter family member
OLR1: oxidized low density lipoprotein receptor 1
PD: pharmacodynamics
PIO: pioglitazone
PK: pharmacokinetics
PKM2: pyruvate kinase
PPARγ: peroxisome proliferator-activated receptor γ
PPRE: peroxisome proliferator response elements
PTPRD: protein tyrosine phosphatase receptor type D
RETN: resistin
ROSI: rosiglitazone
SLCO: solute carrier organic anion transporter family member
SNP: single nucleotide polymorphism
TNFα: Tumor necrosis factor α
TZD: thiazolidinedione
T2DM: type 2 diabetes mellitus
UCP2: uncoupling protein 2.
