# Supplementary figures and images for: Current clinical evidence on pioglitazone pharmacogenomics
Source: Front Pharmacol. 2013 Nov 26;4:147. doi: 10.3389/fphar.2013.00147 (PMC3840328; doi:10.3389/fphar.2013.00147)

## SUPPLEMENTAL FIGURE 1

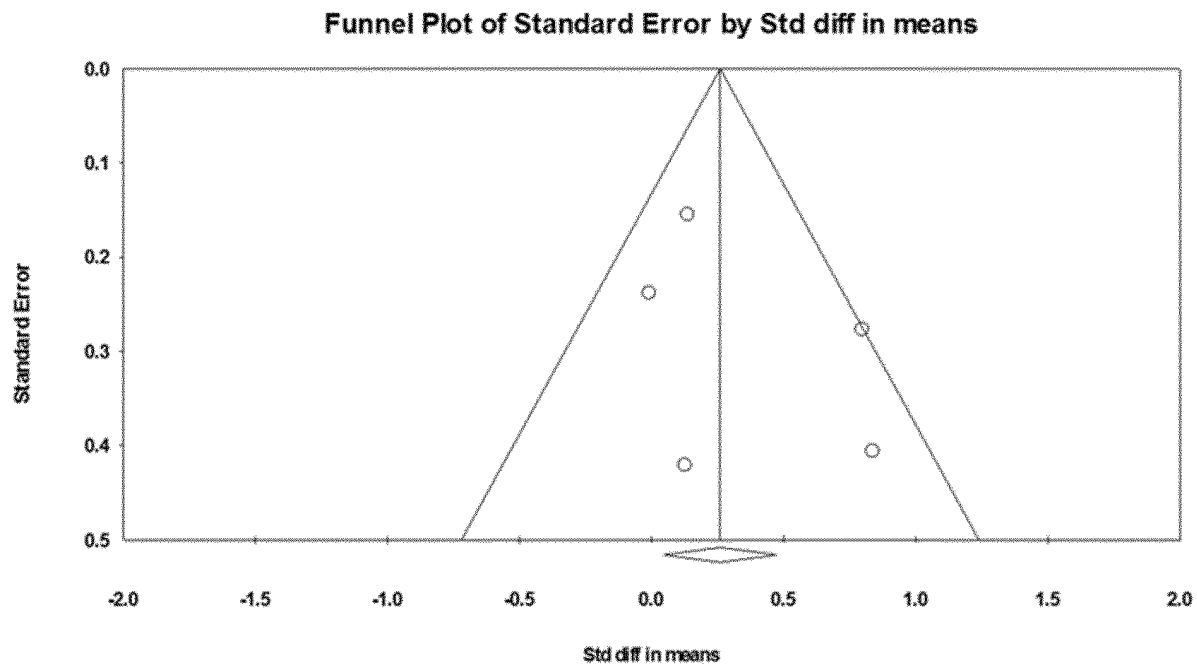

Supplement: Supplementary file 1 [file Presentation1.PDF]
